# Bayesian algorithms for recovering structure from single-particle diffraction snapshots of unknown orientation: a comparison

**DOI:** 10.1107/S0108767311019611

**Published:** 2011-07-06

**Authors:** Brian Moths, Abbas Ourmazd

**Affiliations:** a Department of Physics, University of Wisconsin–Milwaukee, 1900 E. Kenwood Boulevard, Milwaukee, WI 53211, USA

**Keywords:** X-ray scattering, single-particle structure determination

## Abstract

The advent of X-ray free-electron lasers promises the possibility to determine the structure of individual particles such as microcrystallites, viruses and biomolecules from single-shot diffraction snapshots obtained before the particle is destroyed by the intense femtosecond pulse. This program requires the ability to determine the orientation of the particle giving rise to each snapshot at signal levels as low as ~10^−2^ photons per pixel. Two apparently different approaches have recently demonstrated this capability. Here we show they represent different implementations of the same fundamental approach, and identify the primary factors limiting their performance.

## Abstract 2

X-ray free-electron lasers are being used to determine the three-dimensional structure of objects from random snapshots. The two apparently very different Bayesian algorithms capable of performing this at ultra-low signal are fundamentally the same.

## 1. Introduction

X-ray free-electron lasers promise to move crystallography beyond crystals. For example, moves are afoot to determine the structure of biological molecules and their assemblies by exposing a succession of individual single particles to intense femtosecond pulses of X-rays (Solem & Baldwin, 1982 ▶; Neutze *et al.*, 2004 ▶; Gaffney & Chapman, 2007 ▶). In addition to experimental issues, two algorithmic challenges must be overcome in order to recover structure from such diffraction snapshots. First, the orientation of the object giving rise to each snapshot must be determined. Second, this must be performed at extremely low signal. A typical 500 kD bio­molecule, for example, scatters only 100 of the ~10^12^ incident photons, with the photon count per pixel being as low as 10^−2^ at the detector (Shneerson *et al.*, 2008 ▶). As the particle orientations giving rise to the snapshots are unknown, the signal cannot be boosted by averaging, and orientation recovery must be carried out at ‘raw signal level’ in the presence of shot (Poisson) and background scattering noise (Shneerson *et al.*, 2008 ▶; Fung *et al.*, 2009 ▶). Orientation recovery is thus one of the most critical steps in single-particle structure determination (Leschziner & Nogales, 2007 ▶). Once diffraction-pattern orientations have been discovered, the three-dimensional diffraction volume can be assembled and the particle structure recovered by standard phasing algorithms (Gerchberg & Saxton, 1972 ▶; Feinup, 1978 ▶; Miao *et al.*, 2001 ▶; Shneerson *et al.*, 2008 ▶; Fung *et al.*, 2009 ▶; Loh & Elser, 2009 ▶).

Using an adaptation of generative topographic mapping (GTM) (Bishop *et al.*, 1998 ▶; Svensén, 1998 ▶), Fung *et al.* (2009 ▶) published the first successful recovery of the structure of a molecule from simulated diffraction snapshots of unknown orientation at signal levels expected from a 500 kD molecule by utilizing the information content of the entire ensemble of diffraction snapshots. Subsequently, Loh & Elser (2009 ▶) demonstrated structure recovery from simulated diffraction snapshots by an apparently different approach, using a so-called expansion–maximization–compression (EMC) algorithm (Loh & Elser, 2009 ▶). Both approaches have been validated with experimental data. Loh *et al.* (2010 ▶) have oriented snapshots from iron oxide nanoparticles obtained by single-shot diffraction. Using GTM, Fung *et al.* (2010 ▶) and Schwander *et al.* (2010 ▶) have determined the orientation of diffraction snapshots from gold nanofoam with ~8 × 10^−2^ scattered photons per Shannon pixel with an orientational accuracy of about one Shannon angle. Using a variety of manifold embedding approaches, Giannakis *et al.* (2010 ▶) have demonstrated orientation recovery from diffraction snapshots of superoxide dismutase crystals with 1° accuracy compared with the goniometer step size of 0.5° and the crystal mosaicity of 0.8°. Using recently discovered symmetries of image formation, Giannakis *et al.* (2010 ▶) have used manifold approaches for orientation recovery and three-dimensional reconstruction of single chaperonin molecules with experimental cryo-electron microscopy snapshots as well as experimental snapshots processed to represent doses 10× lower than is possible with existing techniques.

Here we show the two Bayesian approaches of Loh & Elser (2009 ▶) and Fung *et al.* (2009 ▶) are fundamentally the same, and discuss their capabilities and limitations. Issues to do with the way each approach is implemented and performs under different conditions are beyond the scope of the present paper, if only because these aspects are under active development. In order to facilitate the discussion, the structure-recovery process is divided into two steps: (*a*) orienting the diffraction snapshots and assembling the three-dimensional diffraction volume; and (*b*) recovering the structure by a phasing algorithm. Since we are concerned with orientation recovery, the discussion will be confined to the first step.

The differences in presentation and notation notwithstanding, the Fung *et al.* (2009 ▶) and the Loh & Elser (2009 ▶) approaches are the same in all essential features. Specifically, they both:

(*a*) exploit the information content of the entire data set;

(*b*) recognize that a nonlinear mapping function relates the space of object orientations to the space of scattered intensities;

(*c*) determine the mapping function by Bayesian inference;

(*d*) use the well established expectation–maximization (EM) iterative algorithm (Dempster *et al.*, 1977 ▶) to maximize likelihood;

(*e*) apply a constraint to guide likelihood maximization; and

(*f*) implement noise-robust algorithms with essentially the same computational scaling behaviors.

At the conceptual level, the primary difference between the two approaches concerns the way the step (*e*) is introduced. This paper elucidates the essential similarity between these two approaches, thus clarifying the common basis of Bayesian approaches to orienting snapshots. Details of each approach can be found in the cited references (Svensén, 1998 ▶; Fung *et al.*, 2009 ▶; Loh & Elser, 2009 ▶; Giannakis *et al.*, 2010 ▶). To facilitate a comparison of the two papers, Table 1 ▶ provides a translation table for the symbols used in each.

## 2. Conceptual outline of orientation recovery

In essence, diffraction from a given object is a process (‘a machine’), which takes an orientation as input to generate a diffraction pattern as output. With a detector consisting of *p* pixels, one can represent a diffraction pattern as a vector in a *p*-dimensional Euclidean space of intensities, with the *n*th component of the vector consisting of the intensity recorded at the *n*th detector pixel. The information content of each diffraction pattern can be captured by ensuring that the pixels represent Shannon–Nyquist samples. In this picture, diffraction maps an orientation to a point in a *p*-dimensional space. Because an object has only three orientational degrees of freedom (‘Euler angles’), in the absence of noise, the points in the *p*-dimensional space of intensities define a three-dimensional manifold, which is, in fact, a nonlinear map of the SO(3) manifold of orientations (Giannakis *et al.*, 2010 ▶).^1^

The representation of object orientations bears careful consideration. Despite their widespread use, Euler angles are not a good representation of orientational similarity, because an object can be rotated through large Euler angles (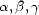) and end at an orientation very close to its starting point. As the Euclidean distance in quaternion space is a good measure of (dis)similarity between orientations, both Fung *et al.* (2009 ▶) and Loh & Elser (2009 ▶) use unit quaternions (Kuipers, 2002 ▶) to represent orientations. Diffraction to a point in reciprocal space, therefore, can be thought of as a functional 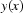, with 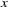 representing a unit quaternion.

A diffraction snapshot consists of *p* intensity values. The mapping thus takes an orientation 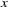 to generate a model snapshot 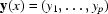. These are to be compared with experimental snapshots 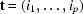, but will, in general, not be identical to any single snapshot owing to (experimental) noise.^2^

Because a given object has only three orientational degrees of freedom, the points 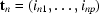 representing the diffraction snapshots in the so-called manifest intensity space trace out a three-dimensional manifold, which is a nonlinear map of the SO(3) manifold of orientations. At a conceptual level, given the ‘input’ and ‘output’ manifolds, it is possible to discover the nonlinear map between them. This links (‘maps’) a diffraction snapshot to a given orientation, and thus assigns an orientation to each diffraction snapshot (Fung *et al.*, 2009 ▶; Giannakis *et al.*, 2010 ▶). Once this has been accomplished, snapshots of similar orientation can be averaged to boost the signal, and structure recovery can proceed by standard techniques. In fact, appropriately wielded, manifold embedding can improve the signal far more efficiently than simple averaging of similar snapshots (Schwander *et al.*, 2010 ▶; Giannakis *et al.*, 2010 ▶), but this is beyond the scope of the present paper.

We now discuss how this conceptual outline forms the basis of the two apparently different approaches by Fung *et al.* (2009 ▶) (hereafter Fung) and Loh & Elser (2009 ▶) (hereafter LE).

## 3. Exploiting the information content of the data set

Both approaches use the conceptual framework that snapshot orientations can be determined by discovering the nonlinear map connecting the two manifolds. The power of this general approach stems from the fact that the intensity manifold is defined by the entire ensemble of snapshots. In essence, one is using the whole data set to assign an orientation to each snapshot. This is needed to overcome the paucity of information in any single snapshot. Key here is the recognition that the ‘mutual information’ between the snapshots of a large ensemble is much larger than the information in any single snapshot (Fung *et al.*, 2009 ▶; Elser, 2009 ▶).

**Figure 1:**
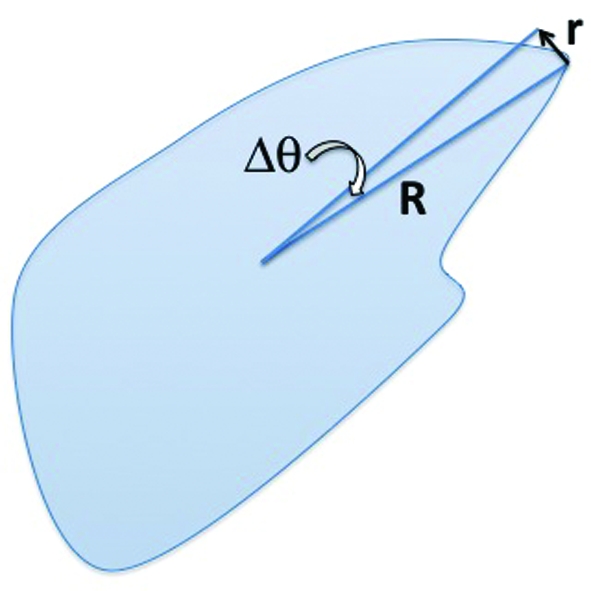
Schematic relationship between object diameter *D* (= 2*R*), spatial reso­lution *r* and required orientational accuracy.

To render the formalism tractable, the SO(3) space of orientations is represented by a discrete set of *K* orientations (‘nodes’) 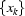, distributed nearly uniformly on the three-sphere (Lovisolo & da Silva, 2001 ▶; Coxeter, 1973 ▶). The inter-node spacing is chosen to satisfy the Shannon–Nyquist sampling criterion, determined as follows. Consider recov­ering the structure of an object with largest diameter *D* (radius *R*) to resolution *r* (Fig. 1 ▶). The orientational accuracy needed is then
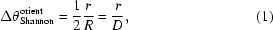
with the number of independent orientations in three dimensions given by
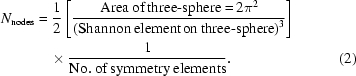
The pre-factor of 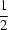 accounts for the fact that the three-sphere is a double-cover of SO(3). The Shannon element in terms of quaternions 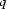 is
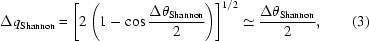
leading to
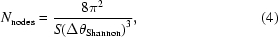
where *S* is the number of symmetry elements of the molecule being reconstructed.

The information content of the data set is compromised by noise. Noise is handled by Fung *via* a Gaussian model for the departures of a vector representing a noisy snapshot from its ideal noise-free position in the *p*-dimensional intensity space. The large number of pixels used as components of a vector representing a snapshot ensures, *via* the central limit theorem (CLT), that a Gaussian model is appropriate regardless of the specific noise spectrum present in each pixel (see Appendix *A* [App appa]). This is important because: (*a*) no prior knowledge of the noise model is required; and (*b*) background scattering, which need not be Poisson in nature, can be dealt with (Schwander *et al.*, 2010 ▶). The use of a Gaussian noise model imposes no restrictions or additional requirements on Fung. LE, at least in its present form, explicitly relies on a Poisson noise model. As pointed out by LE, it remains to be established whether this is sufficient to deal with situations where other types of noise also play a role (Loh & Elser, 2009 ▶).

## 4. Bayesian inference and likelihood maximization

To link the orientations 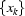 to intensity space, both approaches use Bayesian inference and iterative likelihood maximization. Given a pair of events *A* and *B* with marginal probabilities 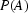 and 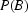, Bayes’ theorem links their conditional probabilities *via* the expression
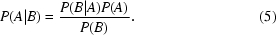
This is used to link the space of orientations with the space of observed diffraction snapshots. Starting with an initial guess for the nonlinear map, the likelihood of the observed data, given the model snapshots 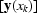, is
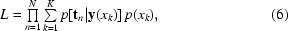
where 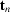 and 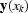 represent the actual and model snapshots, and the indices *n* and *k* run over the set of *N* diffraction patterns and *K* orientations, respectively. The probability 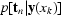 is determined by the noise model, and *p*(*x_k_*) is the prior probability of the orientation *x_k_*, which is 1*/K* when all orientations are equally likely.

Both LE and Fung maximize the log-likelihood iteratively by the well known EM algorithm (Dempster *et al.*, 1977 ▶). Each iteration modifies the model snapshots, effectively moving the manifold defined by them closer to the experimental data. There is no guarantee that the final solution is a global maximum.

Once the mapping corresponding to maximum likelihood has been determined, the orientation of each measured diffraction pattern 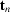 is taken to correspond to that *x_k_* which maximizes the probability of 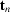 ‘belonging’ to 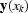. Thus we choose the orientation *x*_*k*_ which maximizes the probability 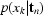. The conditional probability 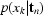 is determined using equation (5).

Having assigned the *N* diffraction snapshots to the *K* orientational bins, the diffraction volume can be reconstructed. In standard ‘classification and averaging’, diffraction patterns assigned to the same orientation *x_k_* are averaged so that there is one representative diffraction pattern for each *x_k_*. So-called generative models such as that used by Fung allow one to construct (‘generate’) model snapshots for each orientation directly from the manifold. As the manifold represents the information content of the entire data set, the generative approach offers significantly greater noise reduction than classification and averaging, which relies on the information in the neighborhood of a given orientation only (Schwander *et al.*, 2010 ▶; Giannakis *et al.*, 2010 ▶).

Each averaged, or alternatively, each generated snapshot is placed in reciprocal space according to its orientation, resulting in a set of irregularly spaced points in reciprocal space. These are interpolated onto a Cartesian grid so as to allow fast Fourier transformation during iterative phasing (Gerchberg & Saxton, 1972 ▶; Schwander *et al.*, 2010 ▶; Feinup, 1978 ▶).

## 5. Constraints to guide expectation–maximization

**Figure 2:**
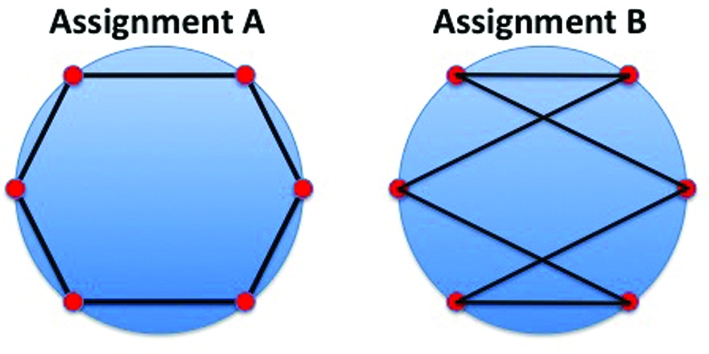
The two different neighborhood assignments indicated by the black lines have the same likelihood. Assignment *A*, which ‘connects’ neighbors, is clearly preferred to assignment *B*. An additional ‘contiguity constraint’ is required to distinguish between these two assignments. The circle perimeters represent the ‘true’ data manifold, the red dots represent the model images 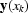 and the black lines represent the neighborhood assignments.

The only substantive difference between the GTM and the EMC algorithms is the way in which the manifold embedding process is introduced, more specifically, the way the model diffraction patterns are evolved so as to maximize the likelihood. In principle, one would modify the model diffraction patterns along the steepest ascent in log-likelihood, until the derivative with respect to changes in the model diffraction patterns is zero. However, this approach is too simple to be of use in practice. Suppose we have found the map *y* such that the likelihood *L* is maximized, and suppose we now exchange a pair of model images assigned to 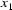 and 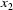, *viz*. 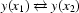. This simply switches the order of the first two terms in the sum over *k* in equation (6), leaving the likelihood unchanged. By the same reasoning, we are able to permute the images assigned to the *x_k_* arbitrarily without changing the likelihood *L*. This means that likelihood maximization alone is unable to find a unique solution, and is, for example, unable to distinguish between the two very different neighborhood assignments shown in Fig. 2 ▶.

In order to eliminate this problem, both the GTM and EMC algorithms place a ‘contiguity constraint’ on the map *y*. This constraint demands that two nodes which are close to each other in the space of orientations be mapped to points close to each other in data space. Fung and LE impose this contiguity constraint differently. In the GTM approach used by Fung, the map is expanded in terms of a set of basis functions:
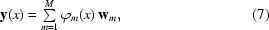
where 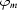 is one of *M* basis functions (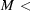 the number of independent orientations *K*) and 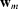 represent the expansion coefficients (weights).

Likelihood maximization proceeds by adjusting the *M* sets of *p* coefficients. The basis functions are chosen so as to vary slowly with *x*. In the current implementation of GTM, they are Gaussians (Bishop *et al.*, 1998 ▶). The map in equation (7) varies slowly, provided the weights 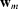 are small. This is achieved by imposing a zero-centered Gaussian distribution on the sum of the squares of the weights. This strategy helps ensure that, topologically, the neighborhood assignments in manifest (intensity) space reflect the neighborhood assignments in latent (orientation) space, *i.e*. 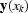 is close to 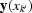 when *x_k_* is close to *x_k′_*.

The EMC algorithm of LE, in contrast, uses the model diffraction patterns 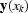 themselves (rather than the weights 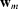) as fitting parameters. After each expectation–maximization step, a so-called ‘compression’ step inserts the model diffraction patterns 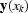 into reciprocal space according to their orientations, and the resulting irregularly spaced points are interpolated onto a uniform grid to determine a new diffraction volume by local averaging. Next, an ‘expansion’ step uses the new diffraction volume as the source for a fresh set of model diffraction snapshots by interpolating back onto the irregularly spaced points corresponding to the pixels of each of the model diffraction patterns. In this approach, both the compression and expansion steps act as low-pass filters; replacing two diffraction patterns by their average and then deducing two diffraction patterns from the average removes sharp variations between diffraction patterns mapped to similar points in reciprocal space. In essence, the so-called compression–expansion cycle is an alternative implementation of the contiguity constraint, whereby neighboring orientations in latent space give rise to neighboring points in manifest intensity space.

The apparently different introductions of the contiguity constraint described above belie the fundamental similarity of the two approaches even in this step. As shown in Appendix *B* [App appb], in the limit of zero weight-regularization parameter in Fung and no compression–expansion in LE, the two approaches reduce to the same algorithm.

## 6. Scaling behavior

The fundamental similarities between the two approaches result in similar scaling in computational behavior. In brief, the computational demands rise as 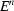, where 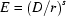 is the number of resolution elements, *D* and *r* the object diameter and spatial resolution, respectively, and *s* the number of orientational degrees of freedom. Typically, 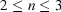, *i.e.* the computational cost scales as the sixth to ninth power of (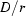) (Fung *et al.*, 2009 ▶), severely limiting the achievable resolution and/or amenable object size. Significant improvements in this behavior are essential, with the most obvious route involving more efficient implementation and parallelization (Fung *et al.*, 2009 ▶; Loh & Elser, 2009 ▶). Fundamentally, however, the high computational cost of Bayesian approaches stems from their generality. It has long been known that the most general algorithms are the most inefficient and the way to improve this involves introducing problem-specific constraints (Le Cun *et al.*, 1990 ▶; Schwander *et al.*, 2010 ▶). This is the basis of a new generation of more efficient algorithms, which directly incorporate the physics of scattering (Giannakis *et al.*, 2010 ▶).

## 7. Summary and conclusions

Bayesian approaches are capable of orienting snapshots containing as few as 100 scattered photons (~10^−2^ photons per pixel). The present paper establishes that two apparently different Bayesian approaches to orienting diffraction snapshots are the same in all essential features. The elucidation of these features can guide the development of computationally more efficient algorithms, which are needed if the large and more complex data sets anticipated from ongoing experiments are to be successfully analyzed. The remarkable capability of the Fung and LE approaches to operate at extremely low signals stems not from algorithmic details, but from the realization that much of the information about a given snapshot resides not in the snapshot itself, but in the other snapshots in the data set, and the entire information content is needed to orient each snapshot at low signal.

## Figures and Tables

**Table 1:** Indices and symbols Translation tables for indices and symbols used in Fung *et al.* (2009 ▶) (Fung) and Loh & Elser (2009 ▶) (LE).

Fung	LE	Description
Indices		
*k*	*j*	Indexes the set of orientations corresponding to the model diffraction patterns
*d*	*i*	Indexes the pixels in an experimental or model diffraction pattern
*n*	*k*	Indexes the set of experimental diffraction patterns
		
Symbols		
**T**	**K**	Matrix whose entries are the pixel intensities of the experimental diffraction patterns
**Y**	**W**	Matrix whose entries are the pixel intensities of the model diffraction patterns
**R**	**P**	Matrix whose entries are the conditional probabilities of the model diffraction patterns, given the experimental diffraction patterns, *e.g. R_kn_* is the probability of the *k*th model diffraction pattern, given the *n*th experimental diffraction pattern

## Footnotes

1 In mathematical terms, diffraction is a mapping , with  describing the diffraction process. In the absence of object symmetry, the map is diffeomorphic, one-to-one and onto. In the presence of object symmetry, the map becomes many-to-one.

2 In this paper, vectors are represented by bold lower-case, matrices by bold upper-case letters.

## Appendix A Gaussian noise model in GTM

The fact that the orientations deduced by GTM agree closely with the correct values for a wide variety of applications, including the case when the noise is strongly Poisson distributed, indicates that the Gaussian model is adequate, at least for the instances we have so far considered. Below we offer a mathematical justification for this observation.

In Svensén’s nomenclature (Svensén, 1998 ▶) GTM maximizes the likelihood function:

where the indices *k* and *n* represent the latent space nodes and the data vectors, respectively. Equation (8) can be written as follows:

The parameter  is a mean, representing the probability of a data vector  belonging to a node, averaged over the  nodes of the manifold. The key point is that the parameters determining the likelihood, and hence the outcome of GTM, are a set of  means. By the central limit theorem (CLT), for sufficiently large , the distribution of  is normal, irrespective of the distributions describing . As  in our case, this is easily satisfied. The normal distribution describing  has mean and variance  independent of the functional form assumed for .

With  and  as fitting parameters, GTM uses the functional form

to fit the data vector cloud. In essence, this is an attempt to describe the data cloud as a sum of Gaussians. As the latter form a complete set, this is permissible, although it may not be the most efficient representation when the noise is Poisson. The CLT is, of course, valid regardless of the representation used to describe the data, and the parameters of the final normal distribution describing  are independent of this choice.

## Appendix B Comparison between contiguity constraint implementations

For GTM, the equation obtained from setting to zero the derivatives of the likelihood with respect to the model parameters is

where the matrix **G** is a *K × K* diagonal matrix with entries given by .

In LE, the model parameters are the pixel intensities themselves, so **Φ** is the identity matrix and **W** = **Y**. There is no weight regularization in the EMC algorithm, *i.e.* λ = 0. Therefore, equation (11) reduces to:

This is to be compared with equation (11) of LE, which, translated into the same notation as equation (12) above, becomes . From the definition of the matrix **G**, it is clear that the LE update rule is given by , which is equivalent to equation (12) above.
